# Conjugation of Coffee Phenolics With Milk Bioactive Peptides: Characterization of Coffee and Apple Phenolics and Evaluation of Polyphenol Oxidase as a Natural Biocatalyst

**DOI:** 10.1002/fsn3.72435

**Published:** 2026-09-30

**Authors:** Bandar N. Hamadneh, Entisar D. Mustafa, Mohamed E. Alkafafy, Ebtihal Khojah, Bassem N. Samra, Daniel Eutyche Mbadjoun Wapet, Mohamed Metwally Mahmoud, Waleed Z. Badawy, Mahmoud Khalil, Mohamed A. Salama, Mohamed Abdin, Mostafa Ali

**Affiliations:** ^1^ Department of Human Nutrition and Dietetics, Faculty of Agriculture Ajloun National University Ajloun Jordan; ^2^ Food Science Department, College of Agriculture Tikrit University Tikrit Iraq; ^3^ Department of Biotechnology, College of Science Taif University Taif Saudi Arabia; ^4^ Department of Food Science and Nutrition, College of Science Taif University Taif Saudi Arabia; ^5^ Department of Biology, College of Science Taif University Taif Saudi Arabia; ^6^ National Advanced School of Engineering Universit'e de Yaound'e I Yaound'e Cameroon; ^7^ ENET Centre, CEET VSB—Technical University of Ostrava Ostrava Czech Republic; ^8^ Jadara University Research Center Jadara University Irbid Jordan; ^9^ Food Technology Department, Faculty of Agriculture Kafrelsheikh University Kafr Elsheikh Egypt; ^10^ Food Industry Technology Program, Faculty of Industrial and Energy Technology Borg Al Arab Technological University (BATU) Alexandria Egypt; ^11^ Nutritional Science Institute Potsdam University Potsdam Germany; ^12^ Nutrition and Food Science Department, Faculty of Home Economics Menoufia University Shibin El Kom Egypt; ^13^ Agricultural Research Center Food Technology Research Institute Giza Egypt

**Keywords:** apple polyphenol oxidase, chlorogenic acid–peptide interactions, coffee beans, MALDI‐TOF‐MS, polyphenol oxidase

## Abstract

This study aimed to characterize phenolic compounds from green coffee beans and apple juices and to evaluate polyphenol oxidase (PPO) from different apple cultivars as a natural biocatalyst for phenolic–milk bioactive peptide conjugation. Phenolic profiling by HPLC–MS analysis showed that chlorogenic acids were the major phenolic compounds in green coffee beans, with 5‐CQA being predominant. Among the tested apple cultivars, Braeburn exhibited the highest PPO activity (6262.4 ± 462.6 U/L) and a moderate chlorogenic acid content. BioZate1 (BZ1) and Glycomacropeptide (GMP) peptides were modified under alkaline and PPO‐mediated conditions. A significant reduction in free CQA levels was observed after 24 h, particularly in alkaline‐treated samples. MALDI‐TOF‐MS and RP‐HPLC analyses provided evidence consistent with the formation of peptide–CQA conjugates, with alkaline modification yielding the highest bound phenolic content (14.95 μg/mg protein for ABZ1 and 10.14 μg/mg for AGMP). These findings indicate that apple‐derived PPO can promote coffee phenolic conversion under the tested conditions and support its potential as a natural biocatalytic system for peptide–phenolic modification. However, the lower CQA‐associated fraction observed with PPO‐mediated treatment compared with alkaline treatment, together with the absence of a heat‐inactivated PPO control, limits definitive attribution of the observed modification specifically to PPO or confirmation of covalent conjugation. Further structural investigations are therefore required to establish the nature and specific sites of peptide–phenolic interactions. Moreover, the developed modification system may provide a basis for future studies on the functional and technological properties of the modified peptides.

## Graphical abstract

Green coffee beans provided a chlorogenic acid (CQA)‐rich extract, while apple juice served as a natural source of polyphenol oxidase (PPO). The coffee phenolics were characterized by HPLC–MS and combined with milk bioactive peptides, BioZate1 and Glycomacropeptide, under alkaline and PPO‐mediated conditions. Following modification, HPLC–MS, MALDI‐TOF‐MS, and RP‐HPLC were used to monitor phenolic conversion, depletion, and peptide–phenolic association. Alkaline treatment showed greater CQA association, whereas apple PPO offers a natural, food‐compatible route for phenolic–peptide modification. The findings support a food‐based strategy for developing peptide–phenolic modified ingredients with potential functional applications.

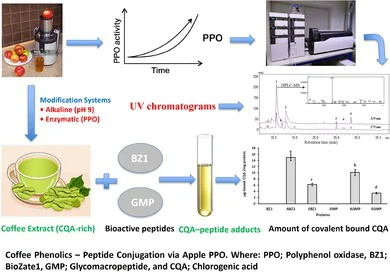

## 1 Introduction

The coffee plant, classified under the Rubiaceae family and *Coffea* genus, comprises more than 100 species, among which *Coffea arabica* and *Coffea robusta* represent the two most commercially important varieties. Coffee is not only one of the most consumed beverages worldwide but also one of the most complex natural matrices, rich in bioactive compounds such as phenolic acids, diterpenes, and alkaloids that contribute to both its sensory properties and health‐promoting effects. Among these compounds, chlorogenic acids (CGAs)—esters of caffeic and quinic acids—constitute the major class of coffee polyphenols, accounting for up to 12% of the bean's dry weight (Awwad et al. 2021; Ali et al. 2023, 2025; Kungsuwan et al. 2023). These phenolics play a critical role in determining the antioxidant potential of coffee and have been associated with several beneficial physiological effects, including modulation of glucose metabolism, protection against oxidative stress, antioxidant, anti‐inflammatory, and anticarcinogenic properties. The primary constituent, 5‐caffeoylquinic acid (5‐CQA), is particularly reactive, capable of undergoing oxidation and isomerization to form bioactive derivatives or quinone intermediates that can covalently interact with biomolecules such as proteins and peptides (Ji et al. 2023; Tarahi et al. 2024; Lešnik et al. 2025).

The use of MALDI‐TOF‐MS and UHPLC‐ESI‐Q‐TOF‐MS profiling can reveal protein profiles and structural alterations in proteins upon conjugation with phenolic compounds (Ali et al. 2013, 2025; Elsebaie and Ali 2022; Elsharkawy et al. 2022). The primary phenolic compounds present in apples include hydroxybenzoic acids, hydroxycinnamic acids, flavanols along with their oligomeric forms (procyanidins) and polymeric forms (proanthocyanidins), as well as flavonols, dihydrochalcones, and anthocyanins. Hydroxycinnamic acids, primarily caffeoylquinic acid, are mainly found in the flesh; flavonols, predominantly quercetin glycosides, are concentrated in the skin; and dihydrochalcones, mainly phloridzin and phloretin glucoside, are the principal compounds of this class. Nevertheless, the levels of these phenolic constituents in apples can vary considerably according to the cultivar, degree of ripeness, and environmental conditions during cultivation (Kschonsek et al. 2018; Hutabarat and Halbwirth 2019; Feng et al. 2021; Arnold and Gramza‐Michalowska 2024). In food chemistry, these phenolic–protein interactions have attracted significant attention due to their capacity to enhance protein functionality, which is a key parameter for functional food applications (Huynh et al. 2026). However, chemical oxidation systems typically used to drive such modifications rely on non‐selective oxidants or high‐pH conditions, which may induce undesirable side reactions, loss of amino acid integrity, or poor control over conjugate formation (Elsebaie and Ali 2022; Cheng et al. 2023; Ke et al. 2023; Baba et al. 2024; Ali et al. 2025).

These drawbacks have motivated the search for natural and enzymatic routes capable of achieving selective, environmentally friendly phenolic conjugation. One particularly promising natural oxidizing catalyst is polyphenol oxidase (PPO), a copper‐containing enzyme widely distributed in plant tissues such as apples, bananas, and mushrooms. PPO catalyzes the two‐electron oxidation of o‐diphenols (e.g., chlorogenic acid or catechol) to their corresponding o‐quinones, which are electrophilic and can react with nucleophilic amino acid residues including lysine, cysteine, tryptophan, and proteins to form covalent adducts (Schilling et al. 2008; Tarahi et al. 2024; Lešnik et al. 2025).

Although chlorogenic acid–protein interactions, using alkaline and enzymatic oxidation systems, have been reported previously, studies investigating the conjugation of coffee‐derived chlorogenic acids with milk bioactive peptides, which differ substantially from native proteins in molecular size, amino acid accessibility, and reactivity, remain scarce. Furthermore, most published studies have employed purified commercial chlorogenic acid and commercial oxidizing enzymes. The present work represents the first stage of a comprehensive study focused on the conjugation of coffee‐derived chlorogenic acids with the milk bioactive peptides BioZate1 and Glycomacropeptide using apple juice‐derived polyphenol oxidase as a natural biocatalyst. Specifically, this study aims to characterize the phenolic composition of green coffee beans and apple juices using HPLC–MS, assess PPO activity among different apple cultivars to identify an optimal biocatalyst source, and evaluate the effect of storage conditions on PPO stability and activity. This approach provides a more realistic food‐based system for developing peptide–phenolic conjugates intended for functional food applications.

## 2 Materials and Methods

### 2.1 Materials

Green Uganda coffee beans were obtained from Rohkaffee‐Company, Berlin, Germany. The fresh apples (Jonagold, Boskoop, Golden Delicious, Cox Orange, and Braeburn) were purchased from a local market in Germany and used as sources of polyphenol oxidase (PPO). Milk bioactive peptides, BioZate 1 (BZ1) and Glycomacropeptide (GMP), were bought from Davisco Foods Company, USA. Chlorogenic acid (CQA) was obtained from Sigma‐Aldrich, Germany.

### 2.2 Methods

#### 2.2.1 Characterization of Phenolic Compounds of Coffee and Apple Juice

##### 2.2.1.1 Extraction of Phenolic Compounds From Coffee Beans

Ground coffee (10 mg) was mixed with 1 mL of a solvent comprising 80% methanol, 19% distilled water, and 1% acetic acid (v/v) and left to extract for 30 min at ambient temperature. After centrifugation (10,000 × g, 30 min, 4°C), the clarified supernatant was transferred to a clean tube and kept at −20°C pending analysis (Ali et al. 2024).

##### 2.2.1.2 Apple Juice Preparation

Fresh apples were washed, quartered, and their cores removed. The apple pieces were then pressed on ice using a fruit juicer (Philips, China). The resulting juice was filtered through a cloth sieve and stored at −20°C until further use.

##### 2.2.1.3 Identification of Phenolic Compounds by HPLC‐MS

Chromatographic analyses were performed on a Shimadzu HPLC system (Kyoto, Japan) equipped with a Prontosil 120–3‐C18 ACE‐EPS analytical column (150 × 4.6 mm, 3 μm; Bischoff Analysentechnik und Geräte GmbH, Leonberg, Germany) and a C18 guard column. The separations were performed at 40°C and a flow rate of 1.0 mL/min, and the UV–Vis spectra were measured in the range of 200–700 nm. Solvent A (2% acetic acid in water) and solvent B (methanol) were used as the mobile phase. Elution was carried out using the following gradient program: 20% B for 3 min, increased to 35% B over 17 min, then to 68% B over the next 17 min, maintained at 68% B for 3 min, returned to 20% B within 3 min and re‐equilibrated at 20% B for 12 min. Total run time was 55 min. Injection volumes were 5–20 μL. Mass spectrometry detection was carried out in positive and negative ionization mode, over a m/z range of 200–1200 Da, with a scan speed of 2000 amu s^−1^ (event time 0.7 s). A nitrogen gas was used for drying at the flow rate of 1.5 L min ^−1^. The interface temperature was 230°C and capillary voltages of 3.0 and 4.5 kV were used for negative and positive ionization modes, respectively. The quantification of chlorogenic acid was performed by external calibration with standard solutions in the range 10–100 μg/mL. Chlorogenic acid concentrations in the samples were calculated from the calibration curve and expressed as μg/mL juice (Ali 2013).

##### 2.2.1.4 Modification of BZ1 and GMP Peptides With Coffee Extract at Different Conditions

BZ1 and GMP peptides were modified using coffee bean extract under two conditions: alkaline (pH 9) and enzymatic (with polyphenol oxidase supplied by apple juice). To prepare the coffee extract, 3 g of ground green coffee was infused in 90 mL of boiling distilled water for 15 min. After cooling to 25°C, the extract was centrifuged at 4000 × g at 4°C for 10 min, and the resulting supernatant was diluted to a final volume of 250 mL. For the alkaline modification, 25 mL of the extract was combined with 0.25 g of GMP and BZ1, and the pH was adjusted to 9. For enzymatic modification, 3 mL of freshly prepared apple juice was added to the reaction mixture, yielding a pH of 4.7. The selected apple juice exhibited a PPO activity of 6262.4 U/L; therefore, approximately 18.8 U PPO were introduced per reaction. Both reaction mixtures were left for 24 h at room temperature (25°C) under continuous stirring and exposure to air. The products were then dialyzed against distilled water at room temperature for 18–24 h, lyophilized, and stored at −20°C until further analysis. Control experiments including peptide‐only incubated under the same reaction conditions, peptide–apple juice mixtures without coffee extract, and coffee extract–apple juice mixtures without peptides were performed (Ali 2013). A limitation of the present study is that a heat‐inactivated apple juice/PPO control was not included. Although peptide‐only, peptide–apple juice without coffee extract, and coffee extract–apple juice without peptides controls were performed, these controls cannot completely distinguish the contribution of PPO from that of other heat‐sensitive or non‐enzymatic components present in crude apple juice. Therefore, future experiments using heat‐inactivated apple juice and/or purified PPO will be necessary to determine the specific contribution of PPO.

#### 2.2.2 Chemical Properties of Modified Peptides

##### 2.2.2.1 Determination of Changes in Free CQA Derivatives

Samples were taken from the reaction mixture at the start and after 24 h to quantify the remaining free CQA derivatives using HPLC, as previously described.

##### 2.2.2.2 Matrix‐Assisted Laser Desorption/Ionization−Time‐of‐Flight−Mass Spectrometry (MALDI‐TOF‐MS) for Peptides

The measurements of peptides were carried out on AUTOFLEX‐III LRF200‐CID, equipped with Smartbeam‐Laser 200 (Bruker Daltonik GmbH) as described in Ali et al. (2013).

##### 2.2.2.3 The Amount of CQA Associated With the Modified Peptides

Reverse‐phase HPLC (RP‐HPLC) analysis was performed on a Shimadzu 10A system (Kyoto, Japan) equipped with a Perfectsil C18 300 ODS column (150 × 4.6 mm; 5 μm) maintained at 37°C. The running conditions were detailed in Ali et al. (2013). Detection was carried out at 325 nm, with external calibration performed using 5‐CQA standards in the concentration range of 10–1000 μg/mL.

#### 2.2.3 Statistical Analysis

All quantitative analyses were performed in triplicate, and each reported value represents the mean ± standard deviation (SD). Statistical analyses were conducted using one‐way analysis of variance (ANOVA) followed by Tukey's post hoc multiple comparison test to evaluate differences among treatments in SPSS software (Version 26), and differences were considered significant at *p* ≤ 0.05.

## 3 Results and Discussion

### 3.1 Identification and Profiling of Individual Phenolic Compounds in Coffee Using HPLC‐MS

The phenolic composition of green coffee beans was investigated using HPLC coupled with UV/Vis detection and mass spectrometry (HPLC–MS). The chromatographic profile at 325 nm (Figure 1) revealed eight major peaks corresponding to chlorogenic acid derivatives. These compounds were identified based on their retention times, UV/Vis absorption maxima, and mass spectral data (Table 1).

**FIGURE 1:**
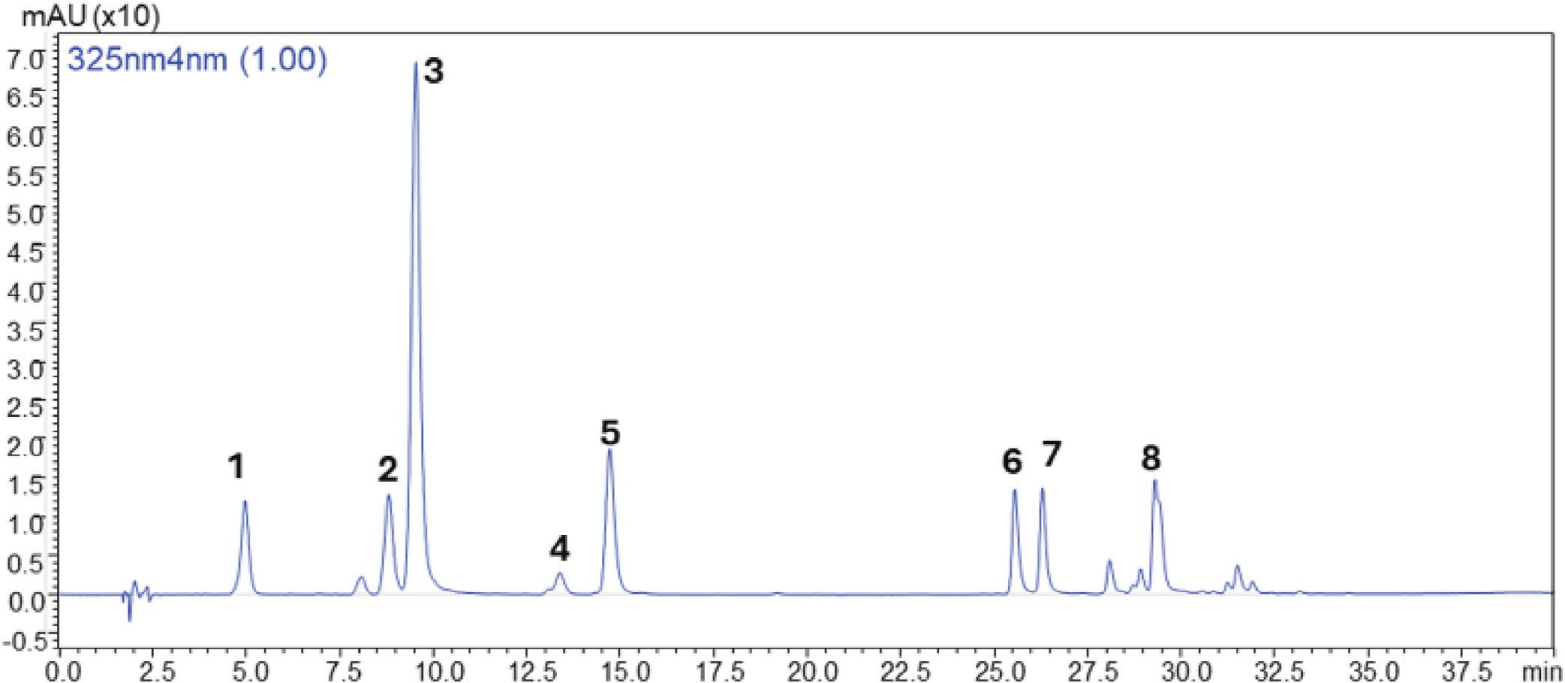
HPLC–MS chromatographic profile of chlorogenic acid derivatives in green coffee beans.

**TABLE 1:** Identification parameters of chlorogenic acid derivatives in green coffee beans.

Peak	1	2	3	4	5	6	7	8
UV/Vis (nm)	280/325	280/325	280/325	280/325	280/325	325	325	325
m/z^a^	353	353	353	367	367	515	515	515
Identity	3‐CQA	4‐CQA	5‐CQA	FQA 1	FQA 2	3,4‐di‐CQA	3,5‐di‐CQA	4,5‐di‐CQA

Abbreviations: CQA, Caffeoyl‐Quinic Acid; di‐CQA, di Caffeoyl‐Quinic Acid; FQA, Feruloyl‐Quinic Acid.

^a^ Affirmed by HPLC‐MS.

The first three peaks (1–3) were identified as caffeoylquinic acid (chlorogenic acid, CQA) isomers, namely 3‐CQA, 4‐CQA, and 5‐CQA, with a common [M–H]^−^ ion at *m/z* 353 and characteristic UV absorption at 280 and 325 nm. Among these, 5‐CQA was the predominant phenolic constituent, showing the highest peak intensity, consistent with previous studies reporting it as the most abundant chlorogenic acid in coffee (Ali et al. 2023, 2025; Kim et al. 2024; Badmos and Kuhnert 2025).

Peaks 4 and 5 were assigned to feruloylquinic acid (FQA) isomers, both (FQA1 and FQA2) exhibiting [M–H]^−^ ions at *m/z* 367. These compounds also showed absorption maxima at 280/325 nm, reflecting the conjugated phenolic structure of feruloyl derivatives. Peaks 6–8 were attributed to dicaffeoylquinic acids (di‐CQAs), including 3,4‐diCQA, 3,5‐diCQA, and 4,5‐diCQA, with [M–H]^−^ ions at *m/z* 515 and absorption maxima at 325 nm. Overall, the chromatographic profile demonstrated that 5‐CQA is the dominant phenolic compound in coffee beans, followed by other CQA isomers, FQAs, and di‐CQAs. The distribution of chlorogenic acid derivatives is in line with previously reported data for Coffea species, confirming that chlorogenic acids represent the major phenolic class in green coffee (Ali et al. 2023; Kim et al. 2024).

### 3.2 Identification of Phenolic Compounds in the Braeburn Apple Variety Using HPLC‐MS

Figure 2 illustrates the exemplary HPLC chromatogram of phenolic compounds extracted from Braeburn apple juice, detected at 275 and 325 nm. Several distinct peaks were observed (Table 2), where the major compounds identified include 5‐O‐caffeoylquinic acid (peak 1, Rt = 10.6 min; [M–H]^−^ = m/z 353) and 4‐O‐p‐coumaroylquinic acid (peak 2, Rt = 12.95 min; [M–H]^−^ = m/z 337), which eluted early and showed strong absorbance, particularly at 325 nm, consistent with their phenolic acid structures.

**FIGURE 2:**
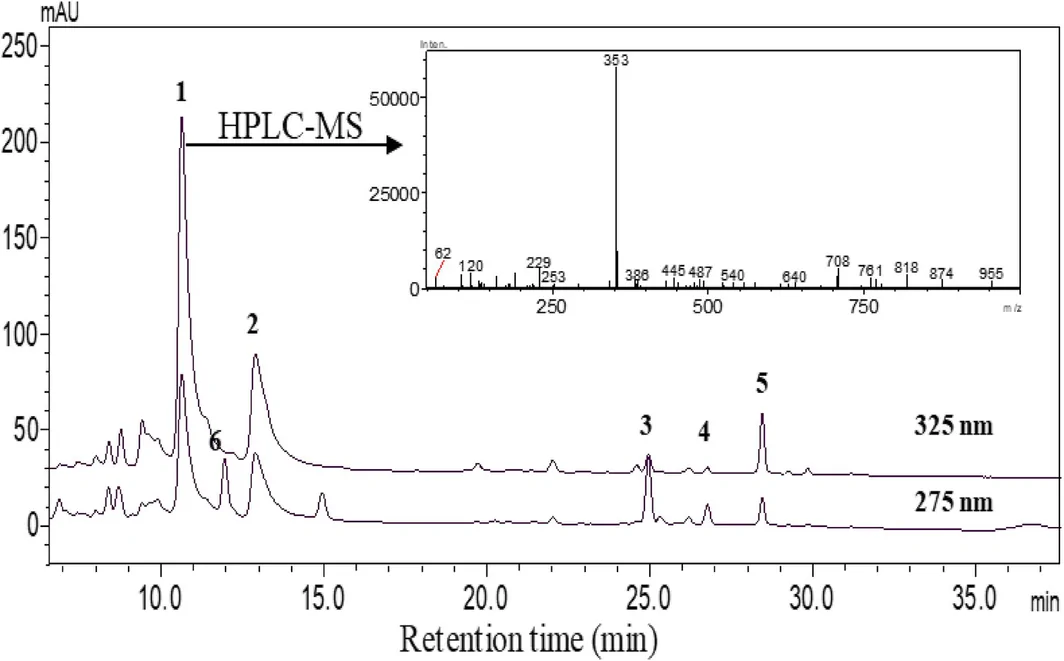
Exemplary UV chromatograms of the phenolics from apple juice (Braeburn variety).

**TABLE 2:** Identification of phenolic compounds in apple juice (Braeburn) using HPLC‐MS.

Peaks	RT (min)	Ionization	Molecular ion	Wavelength	Proposed compound
1	10.60	[M–H]^−^	353	275/325	5‐O‐caffeoylquinic acid
2	12.95	[M–H]^−^	337	275/325	4‐O‐p‐coumaroylquinic acid
3	25.00	[M–H]^−^	567	275/325	Phloretin‐2′‐O‐(2″‐O‐xylosyl) glucoside
4	26.80	[M–H]^−^	754	275/325	Quercetin glycoside
5	28.50	[M–H]^−^	975	275/325	Unidentified
6	12.05	[M–H]^−^	289	275	(+)‐Catechin/Epicatechin

In addition, peak 3 (Rt 25.0 min; [M–H]^−^ m/z 567) was assigned to phloretin‐2′‐O‐(2″‐O‐xylosyl) glucoside. Peak 4 (Rt 26.8 min; [M–H]^−^ m/z 754) corresponded to a quercetin glycoside, tentatively annotated as quercetin‐rhamnose‐hexose‐rhamnose, while an unidentified compound was observed at peak 5 (Rt 28.5 min; [M–H]^−^ m/z 975). Moreover, (+)‐catechin was observed at peak 6 (Rt 12.05 min; [M–H]^−^ m/z 289). All compounds showed absorption at λ_max_ = 275 and 325 nm, except catechin, which showed absorption only at 275 nm (Table 2). These findings align with previously reported results (Marks et al. 2007; Wojdyło and Oszmianski 2020; Teixeira et al. 2023; Dussling et al. 2024).

### 3.3 Characterization of Different Apple Juices

Five fresh apple varieties (Jonagold, Boskoop, Golden Delicious, Cox Orange, and Braeburn) were analyzed for their polyphenol oxidase (PPO) activity and chlorogenic acid (CQA) content to identify the cultivar with the highest PPO activity as the primary criterion for selecting a suitable natural biocatalyst. CQA content was also determined to assess the availability of phenolic substrates in the different cultivars.

### 3.4 The Content of Chlorogenic Acid (CQA)

As described above, the HPLC chromatogram of Braeburn apple juice revealed that CQA was the predominant compound among its phenolic constituents (Figure 2), consistent with previous reports (Markowski et al. 2009; Wojdyło and Oszmianski 2020; Teixeira et al. 2023). Comparison of the five apple varieties showed significant variation in CQA content (Figure 3). The Boskoop cultivar contained the greatest amount of CQA (325.3 ± 5.3 μg/mL juice), while Jonagold ranked next with 141.5 ± 4.6 μg/mL. Cox Orange had the lowest concentration, measuring 63.6 ± 3.2 μg/mL of juice. Such differences are likely linked to varietal genotype, production environment, postharvest practices, maturity at harvest, and storage conditions.

**FIGURE 3:**
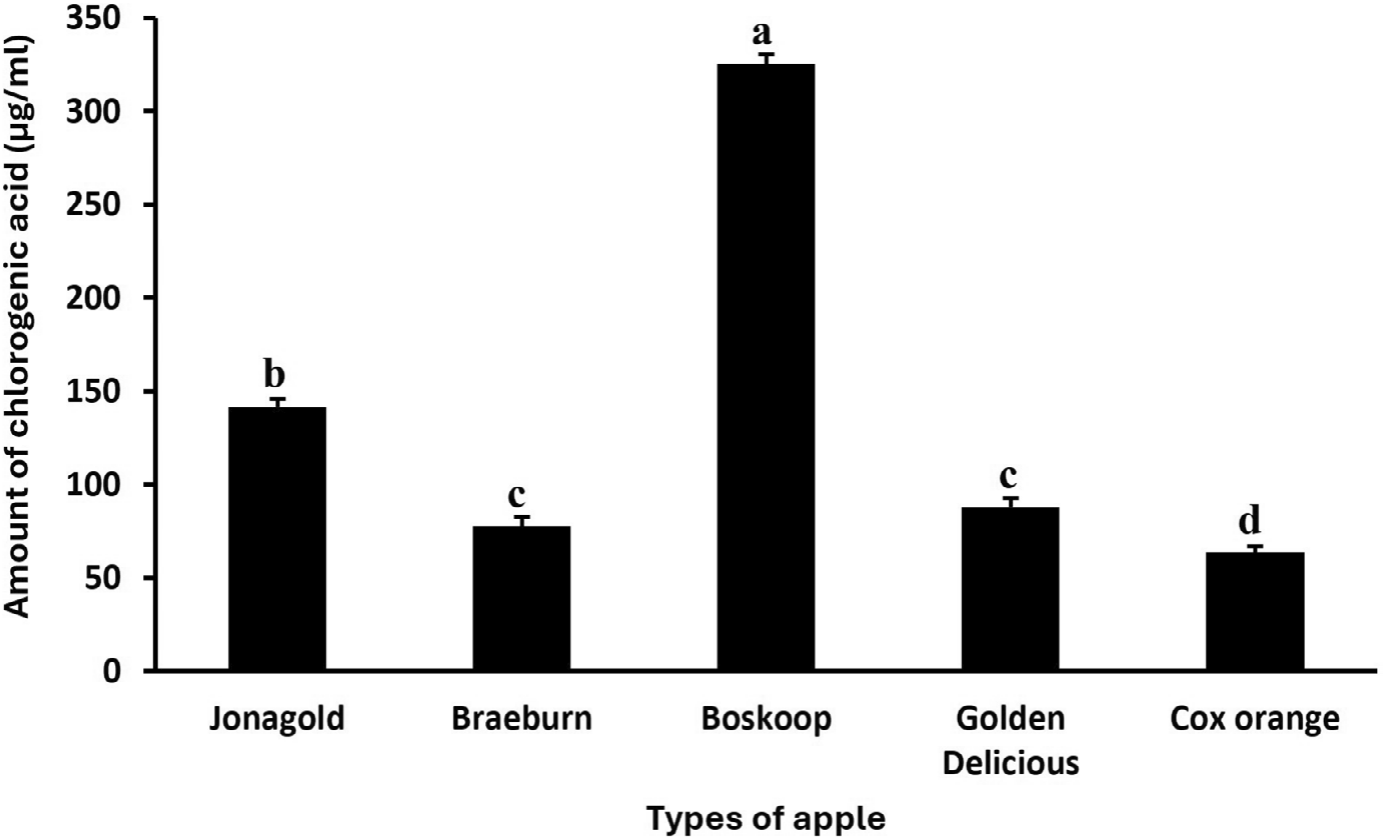
The chlorogenic acid content of different apple varieties. Values are presented as mean ± SD (*n* = 3). Different superscript letters indicate significant differences (*p* ≤ 0.05).

### 3.5 Polyphenol Oxidase (PPO) Activity

Polyphenol oxidase (PPO) activity in apple was quantified by calculating the slope of the linear region of the absorbance–time curve produced by the 4‐methylcatechol (4‐MC)–proline adduct. This adduct forms when phenolic compounds, 4‐MC, and proline react in the presence of apple juice, and the measurement was based on the Lambert–Beer law. Figure 4 illustrates the PPO activity in apple juice samples from different varieties. Figure 4A displays a representative example of the linear increase in absorbance of the formed adduct at 525 nm over time for the Braeburn variety, showing excellent linearity (*R* ^2^ = 0.997), which reflects stable enzymatic activity throughout the reaction period.

**FIGURE 4:**
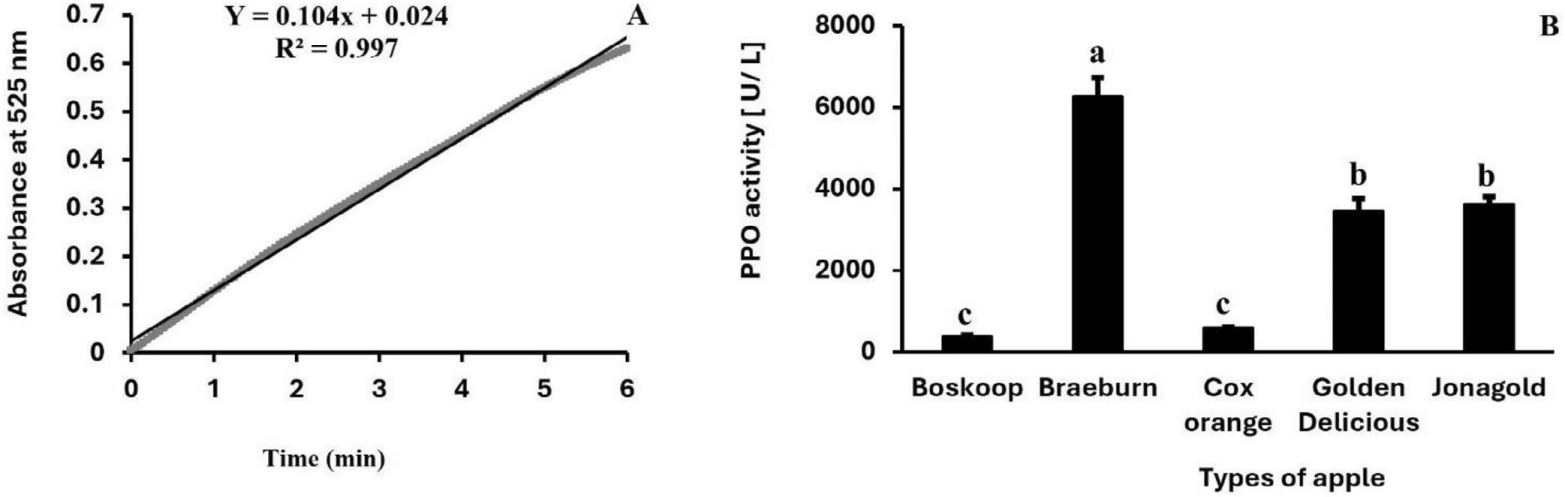
Linear plot of PPO activity for the Braeburn apple variety (A) and PPO activity (U/L juice) across the different apple cultivars (B). PPO refers to polyphenol oxidase. Values are presented as mean ± SD (*n* = 3). Different superscript letters indicate significant differences (*p* ≤ 0.05).

Figure 4B summarizes the PPO activities of five apple varieties, revealing significant differences among them (*p* ≤ 0.05). The Braeburn variety showed the highest PPO activity at 6262.4 ± 462.6 U/L, markedly exceeding that of the other cultivars. Jonagold and Golden Delicious exhibited intermediate PPO activities, with values of 3612.9 ± 186.1 and 3440.9 ± 315.4 U/L, respectively, and no significant difference between them. Conversely, the Boskoop and Cox Orange varieties showed markedly lower PPO activities, with reductions of approximately 94% and 91%, respectively, compared to Braeburn. The observed variation in PPO activity among the apple varieties may be linked to differences in ascorbic acid levels—a natural antioxidant known to suppress polyphenol oxidase activity (McEvily et al. 1992). Additionally, factors such as genetic background, cultivation environment, postharvest treatment, maturity stage, and storage conditions could also contribute to these differences. In summary, Braeburn exhibited the greatest PPO activity among the tested cultivars and was therefore selected as the PPO source for the subsequent enzymatic peptide‐modification experiments. In contrast, Cox Orange exhibited the lowest CQA concentration.

Although Braeburn did not exhibit the lowest chlorogenic acid concentration among the cultivars examined, it showed the greatest PPO activity. Since the primary objective was to identify an efficient natural source of PPO for peptide modification, enzyme activity was considered the most important selection criterion. Moreover, the chlorogenic acid content of Braeburn remained sufficiently high to support enzymatic oxidation reactions. Therefore, Braeburn provided a favorable balance between catalytic potential and substrate availability, making it the most suitable cultivar for subsequent modification experiments.

### 3.6 Influence of Storage Temperature on PPO Activity

Figure 5 illustrates the effect of storage on polyphenol oxidase (PPO) activity in apple juices obtained from fresh Braeburn apples previously stored at different temperatures (25°C, 4°C, −20°C, and −80°C) for 1 day, followed by storage of the extracted juices at −20°C for up to 2 weeks. At week 0, immediately after juice extraction, clear differences were observed among the treatments. Juices prepared from apples stored at −20°C and −80°C showed remarkably high PPO activities (15208.6 and 12163.4 U/L, respectively), whereas those from apples kept at 4°C and 25°C exhibited much lower activities (5209.5 and 1780.7 U/L, respectively). The results indicated significant differences (*p* ≤ 0.05) among treatments, with the highest PPO activity observed in apples frozen at −20°C, followed by those frozen at −80°C. These results indicate that freezing significantly enhances the apparent PPO activity compared to refrigeration or room temperature storage. The increased activity in frozen samples is likely due to ice crystal formation during freezing, which disrupts cellular membranes and releases the membrane‐bound PPO enzyme into the juice matrix, making it more available for catalysis.

**FIGURE 5:**
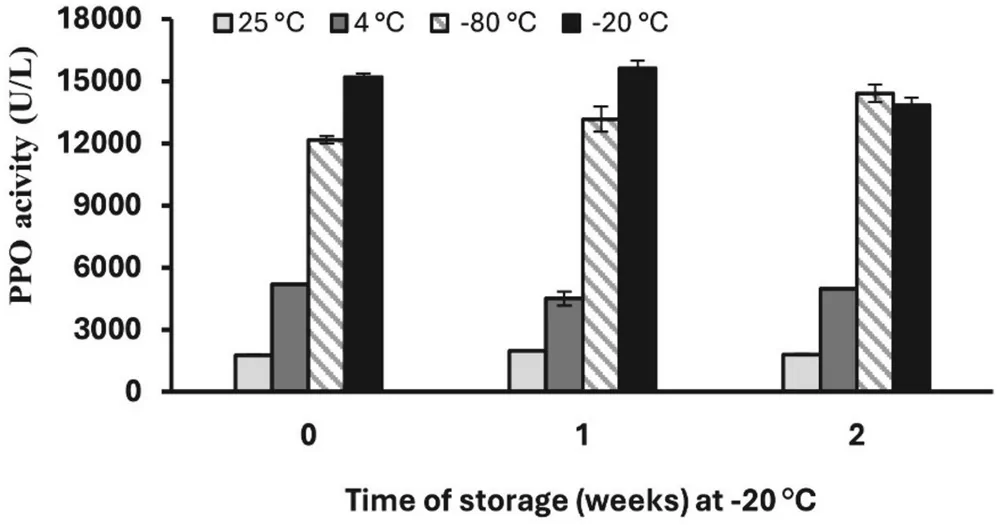
Effect of storage apple juices at −20°C on PPO activity.

After 1 and 2 weeks of storage at −20°C, only minor reductions in PPO activity were observed across all samples, suggesting good enzyme stability under frozen conditions. Juices derived from apples previously frozen at −20°C and −80°C maintained very high PPO activities throughout the storage period, while those from apples stored at higher temperatures remained comparatively low. This stability aligns with the findings of Gouzi and Benmansour (2007), who stated minimal loss of PPO activity after extended frozen storage, highlighting that freezing effectively preserves enzyme functionality in apple juice.

Overall, these results demonstrate that both the pre‐storage temperature of apples and the subsequent frozen storage of juice at −20°C significantly influence PPO activity, with freezing conditions promoting higher and more stable enzymatic activity over time.

### 3.7 The Change in Free CQA Derivatives Within the Solutions Used for the Modification Process

Table 3 summarizes the contents of chlorogenic acid (CQA) derivatives (mg/g protein) in coffee extracts at the start (0 h) and after 24 h of modification using Glycomacropeptide (AGMP, EGMP) and BioZate l (ABZ1, EBZ1). Distinct differences were observed among the samples depending on both the modification system and reaction conditions. At the beginning of the reaction (0 h), 5‐CQA was the predominant isomer across all treatments, ranging between 47.5–51.6 mg/g protein, which was significantly higher than all other CQA derivatives. This observation agrees with previous studies reporting 5‐CQA as the major chlorogenic acid in coffee (Ali et al. 2023, 2025; Behne et al. 2023; Wei et al. 2025). The contents of 3‐CQA and 4‐CQA were considerably lower (7.3–10.8 mg/g protein), while FQA1 and di‐CQAs (3,4‐diCQA, 3,5‐diCQA, and 4,5‐diCQA) were present in minor amounts (< 5 mg/g protein).

**TABLE 3:** Levels of CQA derivatives (mg/g protein) in the coffee extract at the start and completion of the Glycomacropeptide and BioZate1 modification processes.

CQA isomers	Reaction time							
	0 h				24 h			
	AGMP	EGMP	ABZ1	EBZ1	AGMP	EGMP	ABZ1	EBZ1
3‐CQA	9.1 ± 0.1	7.3 ± 0.2	9.5 ± 0.0	7.3 ± 0.2	13.4 ± 0.1	6.8 ± 0.0	9.8 ± 0.1	6.2 ± 0.2
4‐CQA	10.5 ± 0.1	8.2 ± 0.4	10.8 ± 0.0	8.7 ± 0.1	9.6 ± 0.1	7.9 ± 0.0	6.8 ± 0.0	7.2 ± 0.3
5‐CQA	49.4 ± 0.0	51.6 ± 1.4	47.5 ± 0.3	51.1 ± 0.5	17.8 ± 0.4	49.0 ± 1.2	9.5 ± 0.2	45.8 ± 2.1
FQA 1	1.6 ± 0.1	1.2 ± 0.0	1.8 ± 0.0	1.1 ± 0.1	2.3 ± 0.1	1.2 ± 0.1	2.5 ± 0.0	1.3 ± 0.0
FQA 2	9.0 ± 0.0	9.5 ± 0.0	9.0 ± 0.0	8.8 ± 0.7	4.0 ± 0.0	9.2 ± 0.4	2.8 ± 0.1	9.0 ± 0.1
3,4‐di‐CQA	2.9 ± 0.3	2.3 ± 0.0	2.6 ± 0.0	1.9 ± 0.0	0.6 ± 0.0	2.0 ± 0.1	Nd	2.2 ± 0.0
3,5‐di‐CQA	4.6 ± 0.0	3.4 ± 0.2	4.8 ± 0.1	3.2 ± 0.0	0.6 ± 0.0	3.2 ± 0.5	Nd	3.3 ± 0.0
4,5‐di‐CQA	2.5 ± 0.0	2.1 ± 0.2	2.4 ± 0.1	1.6 ± 0.1	0.3 ± 0.0	1.5 ± 0.3	Nd	1.4 ± 0.0
Total‐CQA	69.0 ± 0.2	67.2 ± 2.0	67.8 ± 0.3	67.1 ± 0.8	40.8 ± 0.6	63.6 ± 1.2	26.1 ± 0.3	59.2 ± 2.6
Total‐FQA	10.5 ± 0.1	10.6 ± 0.0	10.7 ± 0.0	9.9 ± 0.8	6.3 ± 0.1	10.5 ± 0.5	5.3 ± 0.1	10.2 ± 0.1
Total‐di‐CQA	10.0 ± 0.3	7.7 ± 0.4	9.8 ± 0.2	6.7 ± 0.1	1.5 ± 0.1	6.8 ± 1.0	0.0 ± 0.0	6.9 ± 0.1

Abbreviations: ABZ1, modified BZ1 with coffee extract at alkaline conditions (pH 9); AGMP, modified GMP with coffee extract at alkaline conditions (pH 9); BZ1, BioZate1; EBZ1, modified BZ1 with coffee extract at enzymatic conditions (3 mL apple juice); EGMP, modified GMP with coffee extract at enzymatic conditions (3 mL apple juice); GMP, Glycomacropeptide; N.d, not detected.

After 24 h, a significant reduction (*p* ≤ 0.05) in total CQA content was observed across all conjugates. For example, AGMP decreased from 69.0 to 40.8 mg/g protein, while ABZ1 showed the greatest decline, from 67.8 to 26.1 mg/g protein. Similarly, total di‐CQAs decreased significantly (*p* ≤ 0.05), particularly in AGMP (from 10.0 to 1.5 mg/g protein). Conversely, the total di‐CQA content in the ABZ1 sample was nearly undetectable. This reduction in CQA could be attributed to its oxidation in alkaline conditions or by the action of polyphenol oxidase, leading to the formation of corresponding quinones (Elsebaie and Ali 2022; Tarahi et al. 2024; Ali et al. 2025). Finally, the type of modification had a significant effect on CQA derivatives. Where, alkaline modified (GMP and BZ1) decreased CQA derivatives levels compared with enzymatically modified (GMP and BZ1). The reduction in 5‐CQA content was more pronounced under alkaline modification compared to enzymatic treatment. This could be attributed to the isomerization of 5‐CQA into its 3‐ and 4‐CQA forms in alkaline conditions. Similar observations have been reported in the literature (Prigent et al. 2008; Schilling et al. 2008). The decrease in free chlorogenic acid derivatives following alkaline and enzymatic treatments should not be interpreted solely as evidence of covalent peptide–phenolic conjugation. Oxidation of chlorogenic acid to reactive quinones may be followed by multiple competing reactions, including peptide conjugation, self‐polymerization, condensation reactions, and structural rearrangements or isomerization. Therefore, the observed reduction in free chlorogenic acid reflects the overall conversion of phenolic substrates within the reaction system rather than a direct measure of covalent binding efficiency alone.

Although alkaline treatment resulted in a greater extent of chlorogenic acid incorporation than the PPO‐mediated system, the enzymatic approach offers several potential advantages. Alkaline conditions are known to promote chlorogenic acid isomerization, non‐specific oxidation reactions, and possible modification of susceptible amino acid residues. In contrast, PPO‐mediated oxidation proceeds under milder conditions and generates reactive quinones through a naturally occurring enzymatic mechanism. Consequently, the enzymatic process may provide greater control over the oxidation reaction while minimizing undesirable side reactions. Moreover, the use of apple‐derived PPO represents a food‐grade and naturally occurring catalytic system that may be more compatible with clean‐label processing strategies than chemical or high‐pH modification approaches.

### 3.8 Effect of CQA on Structural Properties of Peptides

Figure 6 presents the MALDI–TOF mass spectra of the unmodified BZ1 peptide and its modified forms obtained after interaction with coffee extract under two different conditions, alkaline (ABZ1) and enzymatic (EBZ1).

**FIGURE 6:**
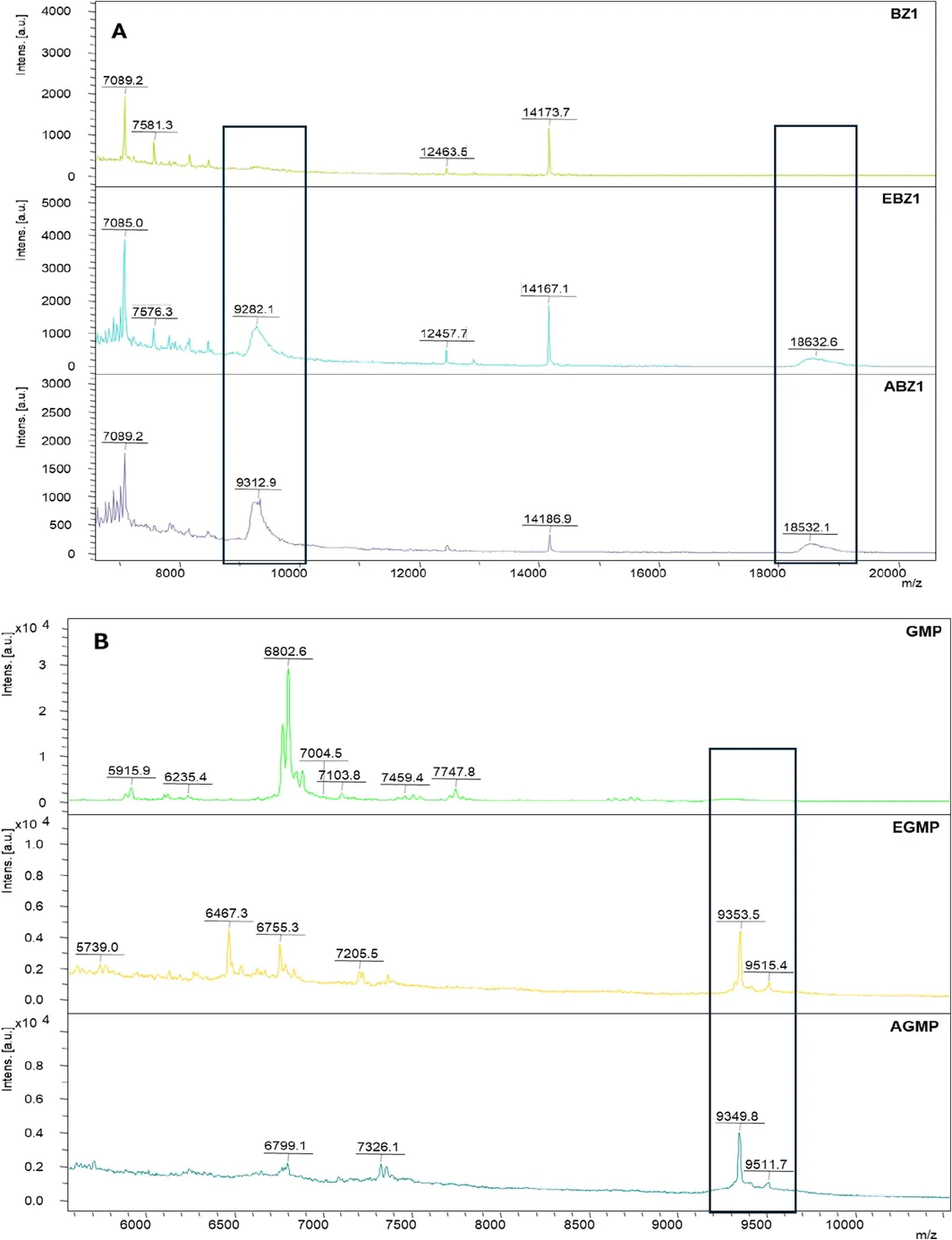
Mass spectrometric analysis (MALDI–TOF) of BioZate1 (A) and Glycomacropeptide (B). Where: BZ1 is unmodified BZ1; ABZ1 is modified BZ1 with coffee extract at alkaline conditions (pH 9); EBZ1 is modified BZ1 with coffee extract at enzymatic conditions (3 mL apple juice); GMP is unmodified GMP; AGMP is modified GMP with coffee extract at alkaline conditions (pH 9); EGMP is modified GMP with coffee extract at enzymatic conditions (3 mL apple juice).

The spectrum of the native BZ1 peptide shows distinct and intense peaks at *m/z* 7089.2, 7581.3, 12463.5, and 14173.7, representing its characteristic molecular ions and confirming the structural integrity of the unmodified peptide. Upon modification, the enzymatically and alkaline treated samples exhibited additional peaks at *m/z* ⁓9300.1 and 18,600. The appearance of these additional peaks indicates substantial molecular modification of BZ1 following treatment with the coffee extract. The observed mass shifts are consistent with the incorporation of coffee‐derived phenolic compounds and/or other modification processes, but they do not by themselves establish the exact chemical nature or covalent binding sites of the products. Moreover, these patterns indicate that the alkaline environment promoted more heterogeneous modification, possibly involving oxidation or random coupling reactions that led to partially degraded or cross‐linked peptide species.

Concerning GMP, Figure 6 illustrates the MALDI–TOF mass spectra of unmodified GMP and GMP modified with coffee extract under the different conditions. The unmodified GMP spectrum shows an intense and sharp peak at m/z 6802.6. After the interaction, remarkable spectral changes were observed. In both modification conditions, two new peaks appeared at m/z ⁓9300 and 9500. The appearance of these additional peaks indicates substantial molecular changes following treatment with the coffee extract. These changes are consistent with the formation of peptide–phenolic products in the presence of apple juice, resulting in the formation of GMP–polyphenol complexes with higher molecular masses. However, MALDI‐TOF‐MS alone does not unequivocally distinguish covalent attachment from other possible modification processes such as cross‐linking or aggregation.

The MALDI‐TOF‐MS results provided preliminary evidence of molecular changes following alkaline and PPO‐mediated treatments, as reflected by the appearance of new mass signals and shifts in peptide mass distributions. However, these spectra were primarily used to demonstrate the occurrence of interactions between peptides and phenolic compounds rather than to establish the exact molecular structures of the resulting products. Consequently, the observed mass changes may reflect a combination of chlorogenic acid incorporation, peptide cross‐linking, aggregation, or other modification reactions. Further structural elucidation using complementary analytical techniques is required to identify specific binding sites and reaction products. Such investigations are currently being addressed in separate studies dedicated to the detailed characterization of the generated conjugates.

### 3.9 The Amount of CQA Associated With the Modified Peptides

Phenolic compounds can interact with proteins through two main mechanisms, covalent and non‐covalent binding (Yilmaz et al. 2022; Shahidi and Dissanayaka 2023; Lu et al. 2025). Under the applied conditions, hydroxycinnamic acid derivatives are oxidized to reactive and redox‐active o‐quinones, which act as electrophilic species capable of reacting with nucleophilic sites on peptides, leading to covalent modification of the peptide structure. An approximate assessment of the modification extent (CQA‐associated fraction, expressed as μg/mg protein; Figure 7) can be obtained using the RP‐HPLC method, which also serves as a useful approach for evaluating the quality of the modified peptides. To eliminate non‐covalently bound CQA, 8 M urea was applied during the analysis. Treatment with 8 M urea was intended to disrupt non‐covalent interactions, including hydrogen bonding and hydrophobic associations. The findings revealed that both free CQA and the CQA–peptide adducts absorbed at the same wavelength (325 nm), but they were distinguished by their different retention times, while the control peptides displayed no significant absorbance at 325 nm. Figure 7 provides evidence consistent with the association of chlorogenic acid derivatives with the peptide matrices, BioZate1 (BZ1) and Glycomacropeptide (GMP), following treatment. The use of 8 M urea was intended to disrupt non‐covalent interactions; therefore, the remaining CQA‐associated fraction is considered consistent with covalent modification. Nevertheless, the RP‐HPLC results do not identify the specific chemical linkage or amino acid residue involved. The degree of CQA–protein association was significantly affected by the applied treatment (*p* ≤ 0.05).

**FIGURE 7:**
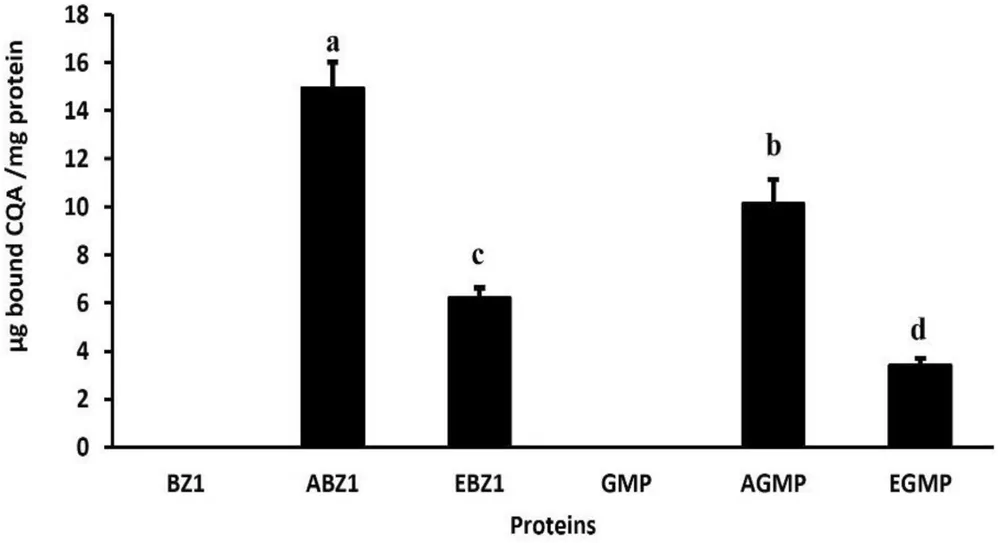
Amount of CQA‐associated fraction in BioZate1 (BZ1) and Glycomacropeptide (GMP) (μg/mg protein). ABZ1 is modified BZ1 with coffee extract at alkaline conditions (pH 9); AGMP is modified GMP with coffee extract at alkaline conditions (pH 9); BZ1 is unmodified BZ1; EBZ1 is modified BZ1 with coffee extract at enzymatic conditions (3 mL apple juice); EGMP is modified GMP with coffee extract at enzymatic conditions (3 mL apple juice); GMP is unmodified GMP. Values are presented as mean ± SD (*n* = 3). Different superscript letters indicate significant differences (*p* ≤ 0.05).

Among the tested treatments, ABZ1 showed the highest amount of bound CQA (14.95 μg/mg protein), significantly exceeding that of EBZ1. This enhanced binding in ABZ1 can be attributed to the greater nucleophilicity of lysine and cysteine residues under alkaline conditions, which facilitates stronger covalent interactions with oxidized phenolic intermediates such as CQA‐derived quinones (Ali et al. 2025). A comparable pattern was found in the GMP‐based samples, where AGMP displayed markedly higher CQA binding (10.14 μg/mg protein) than EGMP, indicating a similar alkaline‐promoted conjugation effect as observed in ABZ1.

Although the observed changes in peptide molecular mass and the detection of CQA associated with the peptide fraction after removal of non‐covalently bound phenolics are consistent with peptide–CQA conjugation, these findings should not be considered definitive structural proof of a specific covalent linkage. MALDI‐TOF‐MS and RP‐HPLC provide supportive evidence for peptide–phenolic modification but cannot unambiguously determine the exact conjugation site or chemical structure of the resulting adducts. Definitive identification of the covalent attachment sites would require complementary high‐resolution LC–MS/MS and peptide‐mapping approaches.

Finally, it should be noted that the alkaline and PPO‐mediated systems were not intended to represent identical reaction environments. The alkaline treatment was included as a reference method commonly used to promote phenolic–protein conjugation, whereas the enzymatic system was designed to simulate food‐relevant conditions using naturally occurring coffee phenolics and apple‐derived PPO. Consequently, the observed differences between treatments reflect not only differences in the oxidation mechanism but also the influence of reaction pH and the overall reaction environment. From an application perspective, the PPO‐mediated system may more closely resemble conditions encountered during food processing and preparation than highly alkaline modification systems.

## 4 Conclusion

The present work demonstrates that conjugation of milk bioactive peptides with coffee‐derived chlorogenic acids can be effectively achieved through both enzymatic (polyphenol oxidase derived from apple juice) and alkaline pathways. Green coffee beans were found to be exceptionally rich in chlorogenic acid derivatives, dominated by 5‐CQA, confirming their suitability as phenolic donors. Among the tested apple varieties, Braeburn exhibited the highest PPO activity and moderate chlorogenic acid content, identifying it as an ideal enzymatic source for controlled conjugation. The enzyme retained strong catalytic activity after freezing at −20°C, ensuring stability for practical use. HPLC–MS analysis further revealed substantial depletion of free CQA isomers following reaction with peptide substrates. During modification, the decline in free CQA content was consistent with oxidation, isomerization, and possible peptide association or conjugation, but did not by itself confirm covalent bonding. Quantitative analysis showed a higher CQA‐associated fraction in alkaline‐treated samples than in enzymatic treatments. The combined use of coffee phenolic extracts and apple‐derived PPO provides a promising natural system for peptide–phenolic modification. The findings establish a foundation for future studies aimed at evaluating the functional, biological, technological, and sensory properties of these conjugates. Additional investigations are required to assess their antioxidant activity, digestibility, bioaccessibility, stability, safety, and potential suitability for food applications.

## Author Contributions

**Bandar N. Hamadneh:** conceptualization, methodology, writing – original draft, supervision. **Entisar D. Mustafa:** conceptualization, methodology, writing – original draft, supervision. **Mohamed E. Alkafafy:** formal analysis, visualization, funding acquisition. **Ebtihal Khojah:** formal analysis, visualization. **Bassem N. Samra:** formal analysis, visualization. **Daniel Eutyche Mbadjoun Wapet:** conceptualization, methodology, software, formal analysis, investigation, data curation, writing – review and editing, validation, project administration, funding acquisition. **Mohamed Metwally Mahmoud:** investigation, data curation, writing – review and editing, validation, project administration, funding acquisition. **Waleed Z. Badawy:** software, investigation, resources. **Mahmoud Khalil:** software, investigation, resources. **Mohamed A. Salama:** software, investigation, resources. **Mohamed Abdin:** software, investigation, resources. **Mostafa Ali:** conceptualization, methodology, writing – original draft, supervision.

## Funding

This research was funded by the Deanship of Graduate Studies and Scientific Research, Taif University.

## Ethics Statement

The authors have nothing to report.

## Consent

The authors have nothing to report.

## Conflicts of Interest

The authors declare no conflicts of interest.

## Data Availability

The data that support the findings of this study are available from the corresponding author upon reasonable request.
